# Vitexin attenuates gastric precancerous lesions with EGFR as a potential target based on network pharmacology and molecular simulations

**DOI:** 10.1038/s41598-026-48952-y

**Published:** 2026-04-23

**Authors:** Jiaying Liu, Nan Liu, Yiting Ye, Zhandong Ye, Dongxu Jiang, Song Huang

**Affiliations:** 1 School of Pharmaceutical Sciences, Guangzhou University of Chinese Medicine, Guangzhou, 510006 Guangdong People’s Republic of China; 2 Shenshan Medical Center, Sun Yat-Sen Memorial Hospital, Sun Yat-Sen University, Shanwei, 516621 Guangdong People’s Republic of China

**Keywords:** Vitexin, Precancerous gastric lesions, EGFR, Epithelial-mesenchymal transition, Migration, Invasion, Molecular docking, Anticancer agent, Cancer, Cell biology, Computational biology and bioinformatics, Drug discovery, Oncology

## Abstract

Precancerous gastric lesions (PLGC) represent a critical transitional stage toward gastric cancer, yet effective interventions to halt progression remain limited. Vitexin, a naturally occurring flavonoid, exhibits anti-cancer potential; however, its molecular targets and mechanisms in PLGC have not been fully defined. This study aimed to elucidate the targets and mechanisms of vitexin against PLGC using an integrative strategy combining network pharmacology, molecular docking/molecular dynamics (MD), and cellular validation. Putative targets of vitexin and PLGC-associated genes were intersected to obtain 33 common targets, followed by protein–protein interaction network construction and pathway enrichment analyses. Molecular docking was performed against core targets, and MD simulations were conducted to assess the stability of the EGFR–vitexin complex. In vitro validation was carried out in MNNG-induced MC cells using cell viability, migration, and invasion assays, phase-contrast imaging, and immunofluorescence staining of EGFR and E-cadherin. Network analysis highlighted CCND1, EGFR, and ABL1 as central nodes and implicated oncogenic programs including cell-cycle regulation and PI3K–Akt signaling pathway. Docking suggested favorable binding of vitexin to the core targets. MD simulations further supported a stable binding mode of vitexin within the EGFR pocket over 100 ns with preserved global compactness; a late-stage conformational adaptation accompanied by hydrogen-bond network reorganization was observed without signs of global unfolding. Functionally, vitexin reduced MC cell viability in a dose-dependent manner, reaching ~ 50% inhibition at 20 μM, and significantly suppressed cell migration and invasion. Immunofluorescence demonstrated decreased EGFR expression and altered membrane localization, together with restored E-cadherin expression and membrane localization, suggesting a shift toward an epithelial phenotype. Vitexin inhibits PLGC-associated malignant phenotypes through multi-target modulation. Among the identified candidates, EGFR may serve as a putative and functionally relevant target, while modulation of EGFR-associated signaling together with restoration of E-cadherin may contribute to the reversal of EMT-related phenotypes. These findings provide mechanistic and structural support for the further preclinical evaluation of vitexin in preventing gastric cancer progression.

## Introduction

Gastric cancer (GC) ranks as the fifth most prevalent cancer globally and stands as the third leading cause of cancer-related mortality, underscoring the urgent need for effective interventions to curb its progression^1^. The development of GC is a complex, multistep process influenced by a confluence of genetic, environmental, and lifestyle factors^2^. Precancerous gastric lesions (PLGC) represent a pivotal stage in this progression, marking a critical juncture where early intervention could significantly mitigate the risk of gastric cancer development^3^. PLGC encompasses a spectrum of pathological changes, including intestinal metaplasia (IM) and dysplasia (DYS), which harbor the potential for malignant transformation^4^. These lesions often arise in the context of chronic atrophic gastritis, influenced by Helicobacter pylori infection and other factors, with the risk of cancer escalating in tandem with the severity of the precancerous changes^5^. Consequently, PLGC presents a crucial therapeutic target, offering a window of opportunity to arrest the carcinogenic process.

Epithelial-mesenchymal transition (EMT) is a fundamental biological process wherein epithelial cells undergo a phenotypic transformation, losing their cell polarity and adhesion properties to acquire a more motile and invasive mesenchymal phenotype^6^. This process, while essential for tissue repair and embryonic development, can be aberrantly activated in cancer, contributing to tumor initiation, progression, and metastasis^7^. EMT is orchestrated by a cascade of signaling molecules within the tumor microenvironment, including transforming growth factor-β1 (TGF-β1), tumor necrosis factor-α (TNF-α), and various carcinogens such as N-methyl-N'-nitro-N-nitrosoguanidine (MNNG)^8^. Characterized by the downregulation of epithelial markers like E-cadherin and the upregulation of mesenchymal markers such as N-cadherin and vimentin, EMT facilitates the detachment of tumor cells from their primary site, enabling them to infiltrate surrounding tissues and metastasize^9^. The reversibility of EMT holds significant clinical promise for the early prevention and treatment of gastric cancer.

Vitexin, a naturally occurring flavonoid compound, has garnered considerable attention for its potential anticancer properties. Previous studies have highlighted its antioxidant, anti-inflammatory, and antiproliferative activities, suggesting its therapeutic potential in various cancers^10,11^. However, the specific molecular targets and mechanisms through which vitexin modulates PLGC remain largely unexplored. Given the critical role of PLGC in gastric cancer progression and the reversible nature of EMT, elucidating vitexin’s effects on these processes could provide valuable insights into its potential as a therapeutic agent for preventing gastric cancer.

In this study, we aim to investigate the molecular targets and mechanisms underlying the effects of vitexin on PLGC using an integrative approach that combines bioinformatics, molecular docking, and cellular assays. We hypothesize that vitexin exerts its anticancer effects by targeting key signaling pathways involved in cell proliferation, migration, and invasion, potentially through EGFR-associated signaling, restoration of E-cadherin expression, and reversal of EMT-related phenotypes. Our findings may offer a novel therapeutic strategy for the management of PLGC and contribute to the development of targeted therapies for gastric cancer prevention.

## Materials and methods

### Materials

N-methyl-N'-nitro-N-nitrosoguanidine (Fig. 5A MNNG, purity ≥ 98%, CAS. No. 70–25-7) was purchased from Chroma Biotechnology Co. Ltd. (Chengdu, China), dissolved in DMSO solution and diluted to the corresponding concentration when applied to GES-1. Vitexin (Fig. 5B PubChem CID: 5,280,441, HPLC ≥ 98%) was purchased from Aladdin Chemical Reagent Co., Ltd. (Shanghai, China).

### Collection of chemical and target information on vitexin

Relevant literature was retrieved from CNKI, Wanfang, Weipu, and PubMed using the keywords “Vitexin.”Reported vitexin structures were compared, and the structure was confirmed in the PubChem database (https://pubchem.ncbi.nlm.nih.gov/). Basic chemical information was compiled, the nomenclature was standardized, and the 2-D structure was downloaded and saved. The SDF file of the structure was uploaded to the SwissTargetPrediction server (http://www.swisstargetprediction.ch/) with “Homo sapiens” selected to generate putative targets. Protein names were converted to official gene symbols with UniProt (https://www.uniprot.org/).

### Collection of gastric precancerous lesion (PLGC)–related targets

GeneCards (https://www.genecards.org/) was searched with the terms “precancerous lesions of gastric cancer,” “gastric precancerous lesions,” and “PLGC.” Retrieved gene lists were merged and duplicates removed. Overlapping genes between vitexin targets and PLGC-related genes were identified as potential therapeutic targets and imported into Cytoscape 3.9.0 to construct a “vitexin–target–disease” network.

### Protein–protein interaction (PPI) network construction

To identify core genes, the overlapping targets were submitted to STRING 11.5 (https://cn.string-db.org/). Interactions with confidence scores > 0.4 were retained and isolated nodes hidden. The resulting PPI network was imported into Cytoscape 3.9.0 for topological analysis and visualization to obtain hub targets.

### GO and KEGG enrichment analysis

Hub targets were uploaded to Metascape for Gene Ontology (GO) and Kyoto Encyclopedia of Genes and Genomes (KEGG) enrichment. Significant pathways involved in vitexin action against PLGC were identified, and a “vitexin–pathway–target–PLGC” network was constructed in Cytoscape 3.9.0.

### Molecular docking

The three-dimensional structures of vitexin and selected core target proteins were obtained from PubChem and the RCSB PDB database, respectively. The ligand structure was converted using Open Babel, and receptor structures were preprocessed in PyMOL by removing water molecules, metal ions, and co-crystallized ligands. Molecular docking was performed using AutoDock Vina 1.2.0. Binding affinities ≤  − 5 kcal mol⁻^1^ were considered indicative of favorable binding, and the conformation with the lowest binding energy was selected for visualization and analysis.

### Molecular dynamics simulation

The docked EGFR–vitexin complex with the best binding pose was subjected to a 100-ns molecular dynamics simulation using GROMACS 2025.2. The protein was modeled with the AMBER99SB-ILDN force field, and vitexin was parameterized using the General AMBER Force Field (GAFF). The complex was placed in a cubic box with periodic boundary conditions and solvated with TIP3P water molecules. Electrostatic interactions were treated using the Particle Mesh Ewald (PME) method, and both van der Waals and Coulomb interactions were truncated at 1.0 nm. After equilibration under NVT (310 K) and NPT (1 atm) conditions, a 100-ns production simulation was carried out in the NPT ensemble with a time step of 2 fs. All bonds involving hydrogen atoms were constrained using the LINCS algorithm. RMSD, RMSF, Rg, SASA, and hydrogen-bond analyses were performed to assess the stability of the EGFR–vitexin complex.

### Cell culture

GES-1 cells were obtained from the Shanghai Chinese Academy of Sciences Cell Bank and cultured in RPMI-DMEM medium supplemented with 10% fetal bovine serum and 1% penicillin/streptomycin (Gibco Life Technology) at 37 °C with 5% CO2 in a humidified incubator. After 24 h, the cells were treated with complete medium containing 4 × 10⁻^5^ mol/L MNNG. The MNNG-containing medium was then replaced with RPMI-1640 medium without MNNG for further culture. During this period, many cells detached and died, while the remaining cells were subcultured when they reached 80%-90% confluence. The resulting cell model, MC cells, was used for subsequent experiments starting from the third passage^12–14^. The MNNG-induced MC cell model was used as an in vitro model relevant to PLGC because MNNG is a classical gastric carcinogen capable of inducing abnormal proliferation, phenotypic alteration, and transformation-related changes in gastric epithelial cells^12–14^. Therefore, this model can partially recapitulate key biological features associated with early gastric precancerous progression and has been used in studies of gastric epithelial injury and precancerous transformation.

### Cell viability assay

Cell viability was assessed using the Cell Counting Kit-8 (AbMole, Shanghai, China) to evaluate the effects of MNNG and Vitexin on GES-1 and MC cells. Cells (5 × 10^3 per well) were seeded in 96-well plates and treated with varying concentrations of MNNG (0 to 100 μM) or Vitexin (0 to 160 μM) for 24 h. Following treatment, cell viability was determined according to the manufacturer’s instructions, and the optical density (OD) at 450 nm was measured using a FlexStation 3 multifunctional microplate reader (Molecular Devices).

### Wound healing assay

To assess MC cell migration, a wound healing assay was performed. Cells were seeded at a density of 1 × 10^6^ cells/well in a 12-well plate and allowed to form a monolayer. A scratch was then made in the monolayer using a micropipette tip. After washing with PBS, the cells were treated with DMEM containing 2% FBS and varying concentrations of Vitexin (0, 5, 10, 20 μM). Migration into the wound space was monitored and images were captured at 0 and 24 h using an inverted microscope.

### Migration analysis

Transwell cell migration assays were conducted using 24-well Transwell plates equipped with 8.0 μm permeable supports (Corning, China). Experimental cells, which had been starved, were prepared in a 200 μL cell suspension and added to the upper chamber. The lower chamber contained 500 μL of RPMI 1640 complete medium supplemented with 10% FBS. Following a 24 h incubation at 37 °C, the cells were treated with vitexin at concentrations of 5 μM, 10 μM, and 20 μM for an additional 24 h before staining. The chamber was then fixed with 4% paraformaldehyde (PFA) and stained with 0.1% crystal violet at room temperature for 20 min. Migrating cells to the lower chambers were visualized using a Nikon Eclipse Ni-U microscope equipped with Imaging Software NIS-Elements 4.0 (Nikon, Japan) for visualization. The images were analyzed using ImageJ 1.52j software (National Institutes of Health, USA).

### Invasion assay

Cell invasion was evaluated using a Matrigel-coated transmembrane cell culture chamber. MC cells were deprived of serum for 24 h in serum-free medium and then digested with 0.25% EDTA trypsin. The cell suspension was subsequently treated with serum-free medium containing various concentrations of vitexin (5 μM, 10 μM, and 20 μM) for 24 h. After the treatment, 200 μL of the cell suspension at a concentration of 1 × 10^5^ cells per well was seeded into the upper chamber of the Transwell insert. Meanwhile, 600 μL of complete growth medium containing serum was added to the lower chamber. The cells were then cultured for an additional 24 h. After culturing, the inserts were collected and fixed with methanol for 20 min, followed by drying. The cells were stained with crystal violet for 20 min. The cells remaining in the upper chamber were gently removed with a wet swab. The inserts were then placed under a Nikon Eclipse Ni-U microscope equipped with Imaging Software NIS-Elements 4.0 (Nikon, Japan) for visualization. The images were analyzed using ImageJ 1.52j software (National Institutes of Health, USA).

### Immunofluorescence (IF) staining

The cells were fixed with 4% paraformaldehyde. Subsequently, after permeabilization with 0.05% Triton X−100 and blocking with 5% bovine serum albumin (BSA), the cells were incubated with primary antibodies against EGFR and E-cadherin, both diluted at a ratio of 1:200, at 4 °C overnight. On the following day, fluorescently—labeled secondary antibodies were applied. Finally, the cells were counterstained with DAPI for nuclear staining. Three random images were captured using a confocal microscope (Nikon, Tokyo, Japan). The fluorescence expression of EGFR and E-cadherin was analyzed using ImagePro Plus v.6.0 software (Media Cybernetics Inc., Rockville, MD, USA). The primary antibodies used in this study were as follows: EGFR (21,773-1-AP) and E-cadherin (27,260-1-AP), both obtained from Proteintech (China).

### Statistical analysis

All experiments were performed in triplicate, and n = 3 represents three independent biological replicates. Data are presented as mean ± SD. Comparisons between two groups were performed using Student’s t-test, while comparisons among multiple groups were analyzed by one-way ANOVA followed by Tukey’s multiple comparisons test. Statistical analyses were conducted using GraphPad Prism 8.0, and P < 0.05 was considered statistically significant.

## Results

### Vitexin target screening and network construction in precancerous gastric lesions

**Fig. 1:**
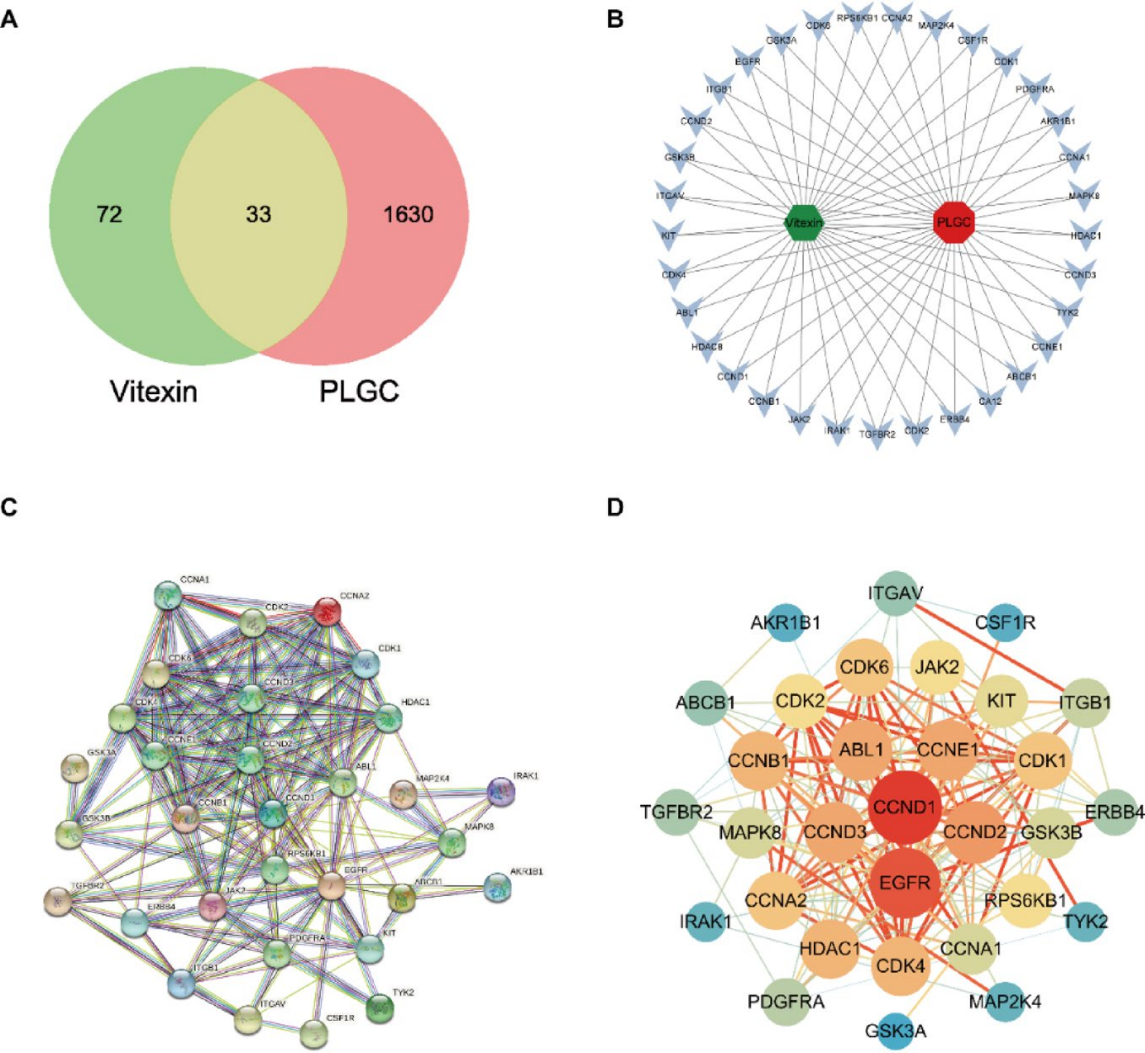
Vitexin target screening and network construction in precancerous gastric lesions. (**A**) Venn diagram of vitexin and precancerous gastric lesion targets. (**B**) Network diagram of vitexin and precancerous gastric lesions. (**C**, **D**) PPI (Protein–Protein Interaction) network diagram.

Figure 1A shows that database screening yielded 105 vitexin targets and 1,663 PLGC-related disease targets. The overlap gave 33 common targets that may mediate vitexin’s effect on PLGC. To see how the active compound fits these targets, we built a “drug–compound–target” network (Fig. 1B). It has a clear hub-and-spoke shape: vitexin sits in the middle, linked to many proteins, illustrating the multi-target nature of herbal medicine.

Next, the 33 shared targets were uploaded to the STRING database to create a protein–protein interaction map (Fig. 1C). The dense connections indicate the proteins do not work alone; they cooperate closely in driving PLGC. Targets with the highest degree values are likely the most important.

Pathway enrichment was then run, and the core target CCND1 was chosen for a detailed map (Fig. 1D). CCND1 occupies the center, directly interacting with EGFR, CDK2, KIT, and others, and in turn regulates downstream genes such as AKR1B1 and ABCB1. The network topology suggests that CCND1 may be an important hub involved in the anti-PLGC effects of vitexin, particularly in relation to cell-cycle regulation.

### Vitexin target GO and KEGG analyses in precancerous gastric lesions

**Fig. 2:**
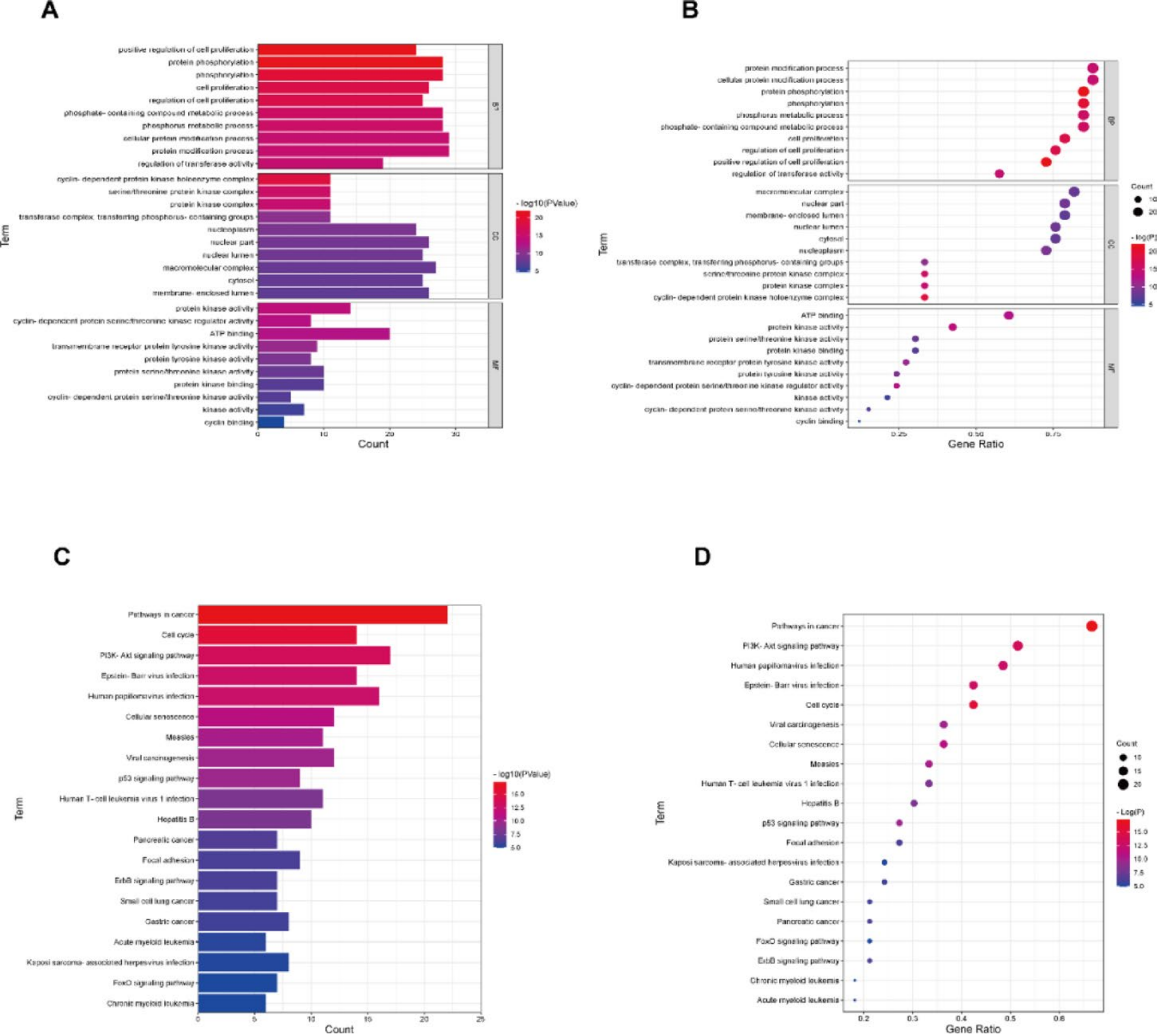
Vitexin target GO and KEGG analyses in precancerous gastric lesions. (**A**, **B**) GO (Gene Ontology) analysis chart. (**C**, **D**) KEGG (Kyoto Encyclopedia of Genes and Genomes) analysis chart.

GO analysis broke the targets into three parts: biological process (BP), cellular component (CC) and molecular function (MF). Figure 2A (bar chart) shows that in BP the hits cluster mainly in “positive regulation of cell proliferation”, “protein phosphorylation” and “regulation of cell proliferation”. For CC, most proteins sit in the nucleoplasm, protein-kinase complexes or cyclin-dependent kinase holoenzyme complexes. MF links them to kinase activity, ATP binding and serine/threonine kinase activity. Overall, these results suggest that vitexin appears to tweak kinase activity, change phosphorylation and slow down growth. Figure 2B (bubble plot) gives the same message. Bubble size = number of genes, colour = –log₁₀(P). Terms such as “protein modification”, “phosphate metabolism” and “cell proliferation” carry both large bubbles and deep red colour, confirming the idea.

KEGG was used to see which pathways matter. Figure 2C (bar chart) lists the top 20. “Pathways in cancer” holds the most genes (23) and the smallest P value (–log₁₀ ≈ 15). Cell cycle, the PI3K–Akt signaling pathway, Epstein–Barr virus infection, p53 signaling pathway, and gastric cancer also scored highly. Thus, vitexin seems to touch cell-cycle control, apoptosis and several cancer routes at once. Figure 2D (bubble plot) adds gene ratio (target genes / background genes). Cancer-related pathways still ranked highest in gene number and significance, while the PI3K–Akt signaling pathway and cell cycle pathway also showed a high gene ratio and strong enrichment signal, indicating that they may represent core pathways involved in the action of vitexin. Taken together, the data suggest that vitexin may affect kinase-related targets in the nucleoplasm, regulate phosphorylation and proliferation-related processes, and inhibit PLGC progression mainly through cancer-related pathways, the PI3K–Akt signaling pathway, and the cell cycle pathway.

### Vitexin–core target binding verified by molecular docking

**Fig. 3:**
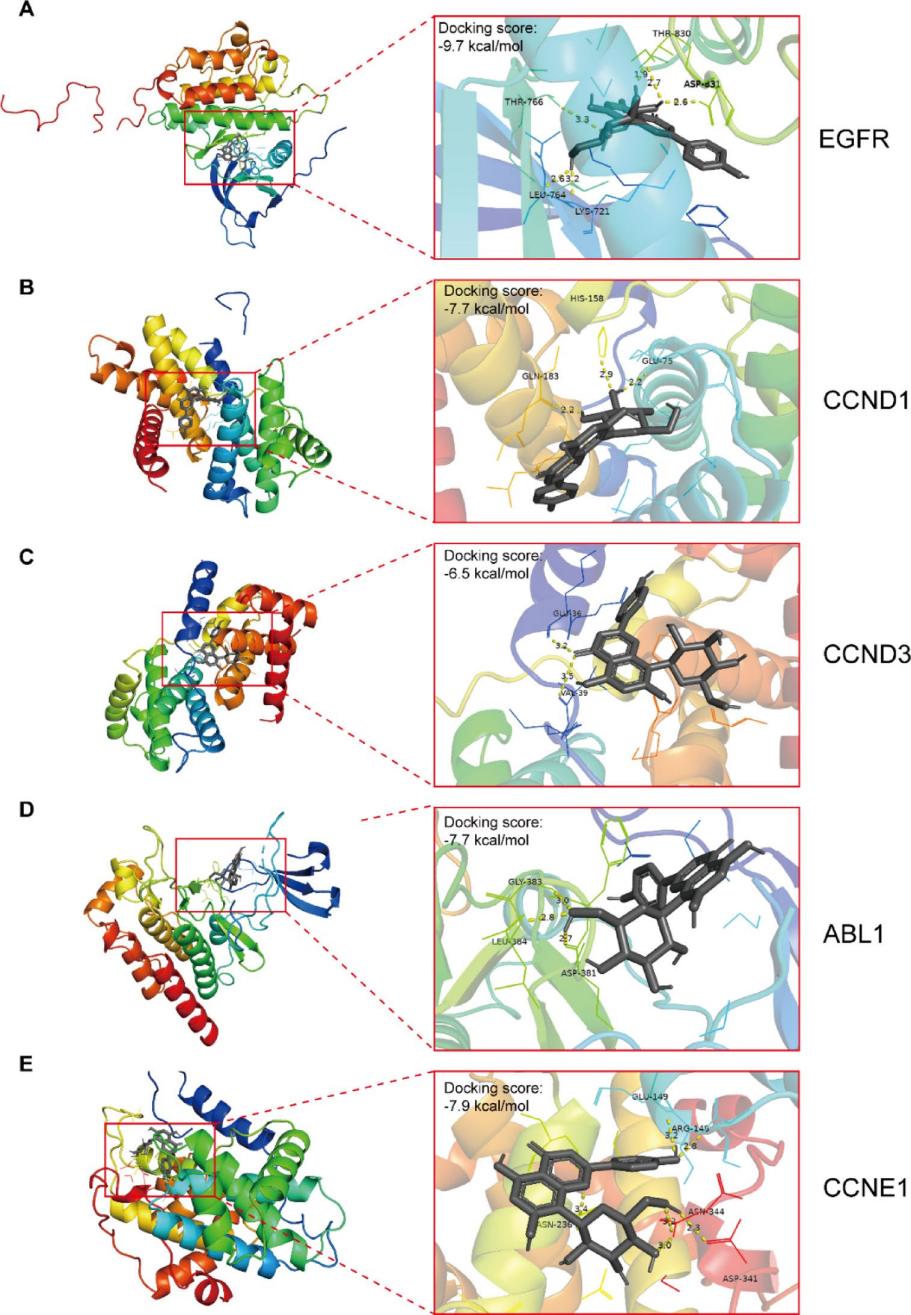
Vitexin–core target binding verified by molecular docking. (**A**) Molecular docking results of vitexin with EGFR target protein. (**B**) Molecular docking results of vitexin with CCND1 target protein. (**C**) Molecular docking results of vitexin with CCND3 target protein. (**D**) Molecular docking results of vitexin with ABL1 target protein. (**E**) Molecular docking results of vitexin with CCNE1 target protein.

To see whether the network predictions hold up and to get a clear picture of how tightly vitexin can grip its top targets, we ran molecular docking on the key nodes from the PPI network. All runs gave binding scores below – 5.0 kcal mol⁻^1^, a cut-off that usually means spontaneous and stable binding (Fig. 3A–E). EGFR gave the best fit, with a score of – 9.7 kcal mol⁻^1^. The ligand slips neatly into the active pocket, making steady contacts with THR-830 and ASP-831 (Fig. 3A). CCND1 and ABL1 came next, both at – 7.7 kcal mol⁻^1^ (Fig. 3B, D). Vitexin anchors to HIS-158 and GLU-75 of CCND1 (Fig. 3B) and to GLY-383, LEU-384 and ASP-381 of ABL1 (Fig. 3D). CCND3 (– 6.5 kcal mol⁻^1^) and CCNE1 (– 7.9 kcal mol⁻^1^) also gave strong poses (Fig. 3C, E). Hydrogen bonds to GLN-136 and VAL-139 stabilize the CCND3 complex, while GLU-149 and ARG-145 pin vitexin inside CCNE1 (Fig. 3E). Taken together, the docking matches the earlier in-silico forecast: vitexin can lodge firmly in EGFR, CCND1, CCNE1 and related proteins that sit at the heart of the cancer and cell-cycle maps. These docking results provide structural support for the potential interaction of vitexin with EGFR, CCND1, CCNE1, and other core proteins, suggesting that these targets may contribute to its inhibitory effects on PLGC-related progression.

### Vitexin exhibits stable binding and interaction profiles within the EGFR complex during a 100-ns MD simulation

**Fig. 4:**
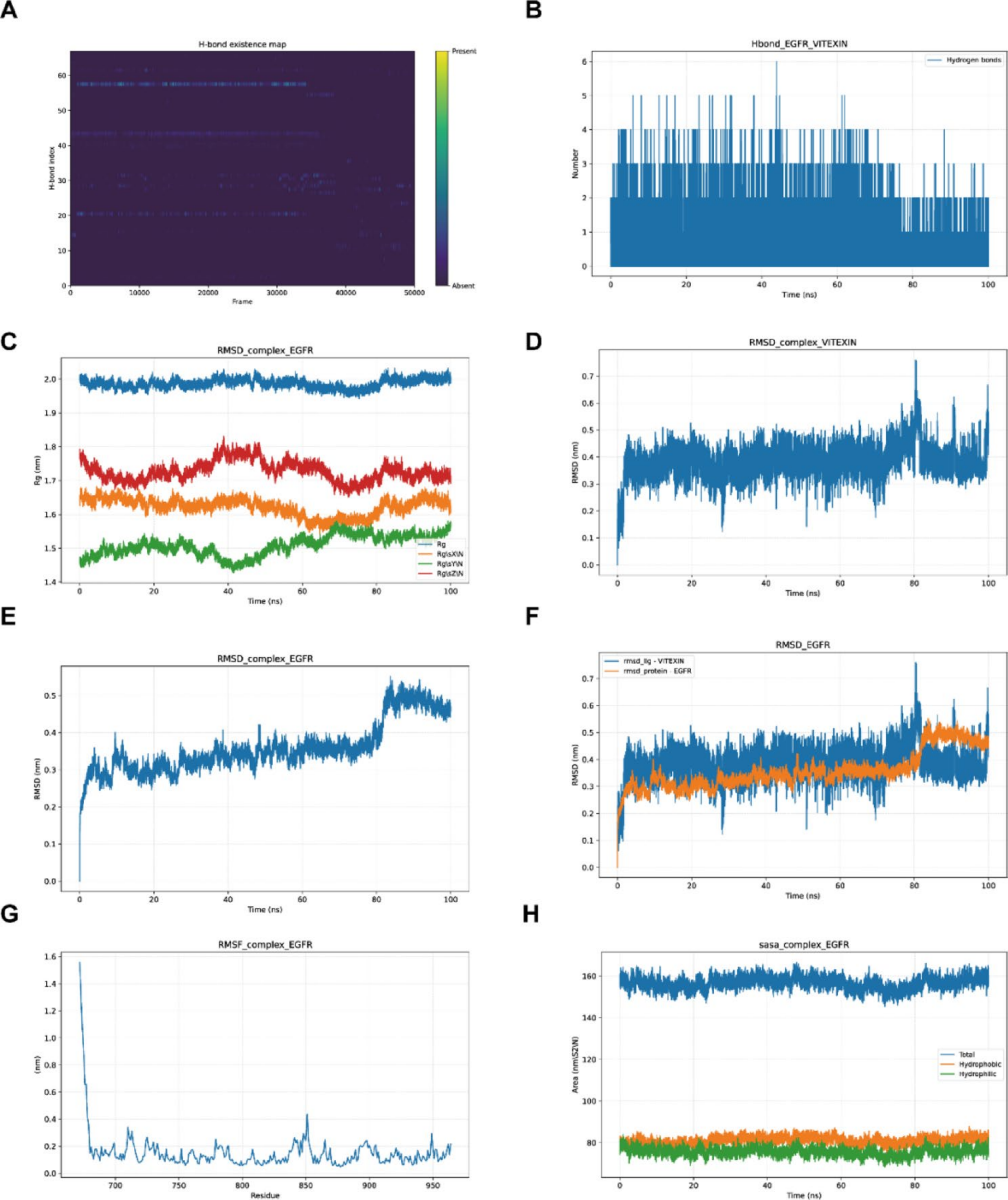
Vitexin maintains stable binding to EGFR while exhibiting a late-stage conformational transition during the 100-ns MD simulation. (**A**) Hydrogen-bond existence map showing the persistence and temporal reorganization of polar contacts between vitexin and EGFR during the simulation. (**B**) Time evolution of hydrogen-bond numbers between vitexin and EGFR. (**C**) Radius of gyration (Rg) of EGFR and its components (RgX, RgY, and RgZ) along the trajectory, reflecting global compactness. (**D**) Ligand RMSD of vitexin relative to the initial binding pose. (**E**) Protein backbone RMSD of EGFR over the simulation time. (**F**) Overlay of ligand RMSD (vitexin) and protein backbone RMSD (EGFR) highlighting the stepwise RMSD transition near ~ 80 ns and the subsequent stable plateau. (**G**) RMSF of EGFR residues, indicating local flexibility with higher fluctuations mainly at terminal regions and a few flexible segments. (**H**) Solvent-accessible surface area (SASA) profiles of EGFR (total, hydrophobic, and hydrophilic components), showing limited changes in solvent exposure.

The protein backbone RMSD reached a relatively stable regime after initial relaxation, fluctuating around ~ 0.30–0.38 nm during 0–80 ns, followed by a stepwise increase to a higher plateau (~ 0.48–0.52 nm) at ~ 80 ns (Fig. 4E, F), indicating a conformational state transition while remaining stable thereafter. Consistently, the ligand RMSD mostly stayed within ~ 0.30–0.50 nm and exhibited a transient spike around ~ 80 ns before returning to baseline fluctuations (Fig. 4D, F), suggesting a short-lived adjustment of the ligand orientation/conformation within the binding pocket rather than persistent drifting.

The global compactness of EGFR was preserved, as the radius of gyration (Rg) remained nearly constant (~ 2.0 nm) without continuous expansion (Fig. 4C). SASA profiles (total, hydrophobic, and hydrophilic components) fluctuated within a narrow range (Fig. 4H), indicating limited changes in overall solvent exposure. Hydrogen-bond analysis showed persistent polar interactions between vitexin and EGFR, with H-bond counts predominantly between 2 and 4 in the early-to-mid stage and more frequently 1–2 in the late stage (~ 75–100 ns) (Fig. 4B). The H-bond existence map revealed several long-lived contacts and a late-stage reorganization of the H-bond network (Fig. 4A). RMSF analysis indicated generally low residue fluctuations (~ 0.10–0.25 nm) except for terminal regions and a few flexible segments (Fig. 4G), supporting an overall stable complex on the simulated timescale. Taken together, the docking and MD simulation results provided structural support for a stable interaction between vitexin and EGFR, thereby supporting the subsequent experimental validation in the MNNG-induced MC cell model.

### Vitexin suppresses the MNNG-induced MC cell model

**Fig. 5:**
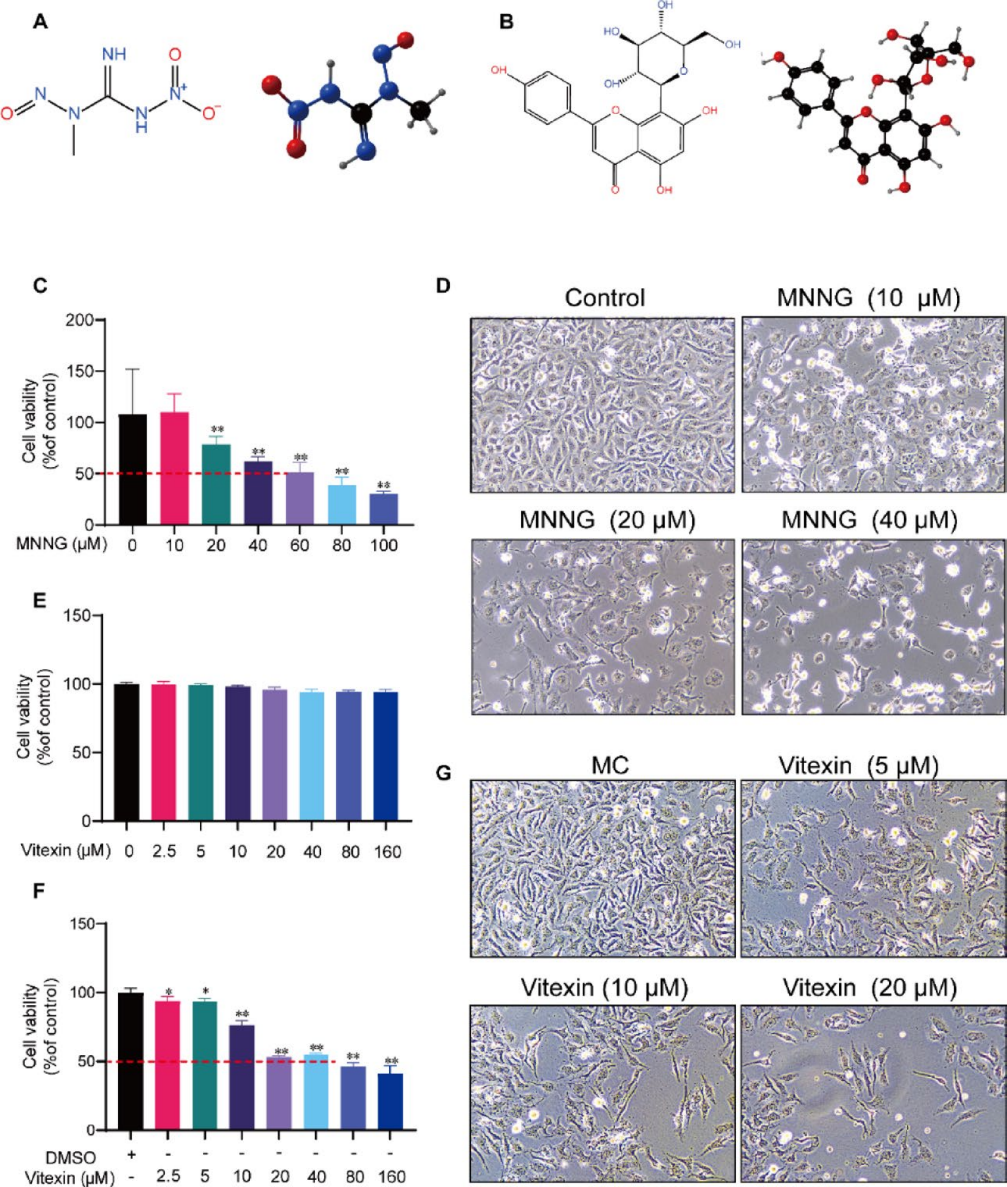
Vitexin suppresses the MNNG-induced MC cell model. (**A**) The 2D and 3D chemical structures of MNNG. (**B**) The 2D and 3D chemical structures of Vitexin. (**C**) The effect of MNNG on the viability of GES-1 cells. (**D**) Morphological images of GES-1 cells after MNNG treatment. (**E**) The effect of vitexin on the viability of GES-1 cells. (**F**) The effect of vitexin on the viability of MC cells. (**G**) Morphological images of MC cells after vitexin treatment. Data are presented as mean ± SD (n = 3). In panel C, *p < 0.05 and **p < 0.01 vs. the untreated GES-1 group. In panel E, *p < 0.05 and **p < 0.01 vs. the untreated GES-1 group. In panel F, *p < 0.05 and **p < 0.01 vs. the MC group.

Figure 5A and B present the chemical structures of MNNG and vitexin. CCK-8 analysis showed that MNNG reduced the viability of GES-1 cells in a dose-dependent manner, with approximately 50% inhibition observed at 40 μM (Fig. 5C, D). Based on this result, 40 μM MNNG was used to establish the MC cell model for subsequent experiments. As shown in Fig. 5E, vitexin did not exhibit obvious cytotoxicity toward normal GES-1 cells within the tested concentration range. In contrast, vitexin reduced the viability of MC cells in a dose-dependent manner (Fig. 5F, G), with approximately 50% inhibition observed at 20 μM. These results indicate that vitexin exerts selective inhibitory effects on MNNG-induced MC cells while showing relatively low toxicity toward normal gastric epithelial cells.

### Vitexin curbs migration and invasion of MNNG-induced MC cells

**Fig. 6:**
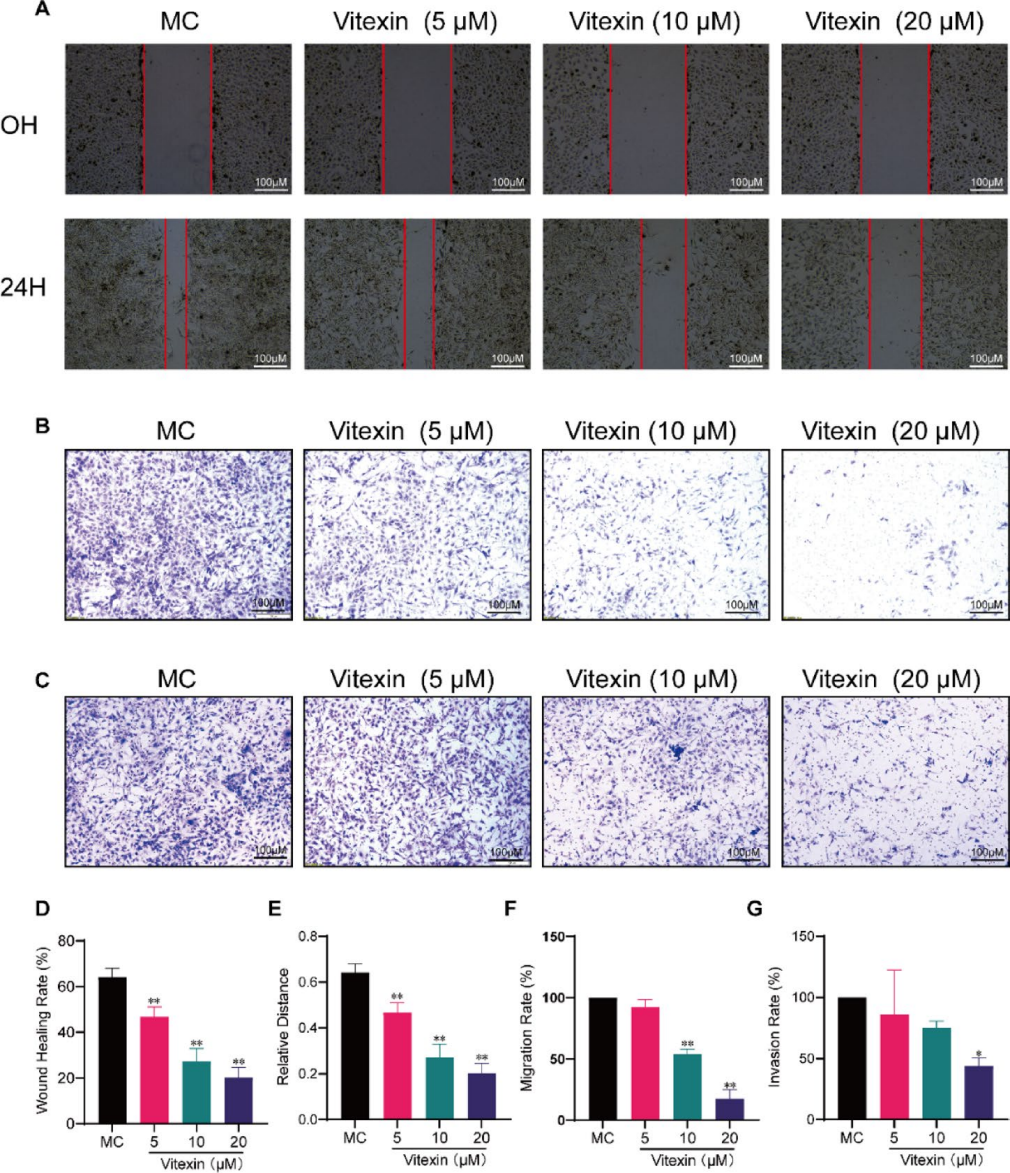
Vitexin inhibits the migratory and invasive abilities of MC cells. (**A**) Representative images of wound healing assays in MC cells treated with vitexin (5, 10, and 20 μM) for 24 h. (**B**) Representative images of Transwell migration assays in MC cells treated with vitexin. (**C**) Representative images of Transwell invasion assays in MC cells treated with vitexin. (**D**) Wound healing rate. (**E**) Relative migration distance in the wound healing assay. (**F**) Migration rate. (**G**) Invasion rate. Data are presented as mean ± SD (n = 3). *p < 0.05, **p < 0.01 vs. the MC group. Scale bar = 100 μm.

Figure 6A, D and E (wound-healing assay) show that after 24 h the scratch in the MNNG-treated MC group had almost closed, confirming the strong migratory phenotype induced by the carcinogen. Vitexin slowed this closure in a clear dose-dependent manner: at 5, 10 and 20 μM the gap remained progressively wider, pointing to a concentration-restrained lateral migration.

Transwell tests gave the same message. In Fig. 6B, F (migration assay) the MC group sent abundant cells through the membrane, whereas vitexin cut the number of migrants stepwise with increasing dose. Figure 6C, G (invasion assay) mirrors this trend; even when a Matrigel coat was added, vitexin again reduced the invading population in a concentration-dependent fashion. Together, the wound-healing, migration and invasion data demonstrate that vitexin can curb both the motility and invasiveness of MNNG-triggered MC cells, and the effect scales with drug concentration. These functional findings add cellular-level support to the idea that vitexin interferes with gastric precancerous progression.

### Vitexin promotes epithelial phenotypes and modulates EGFR-associated signaling in MC cells

Based on the network pharmacology analysis, PPI network screening of core targets, and the molecular docking results, EGFR was selected for further experimental validation. To further investigate the molecular mechanism by which Vitexin inhibits the malignant phenotype of MNNG-induced MC cells, we assessed the expression and subcellular localization of key proteins involved in EMT and oncogenic signaling using immunofluorescence staining.

**Fig. 7:**
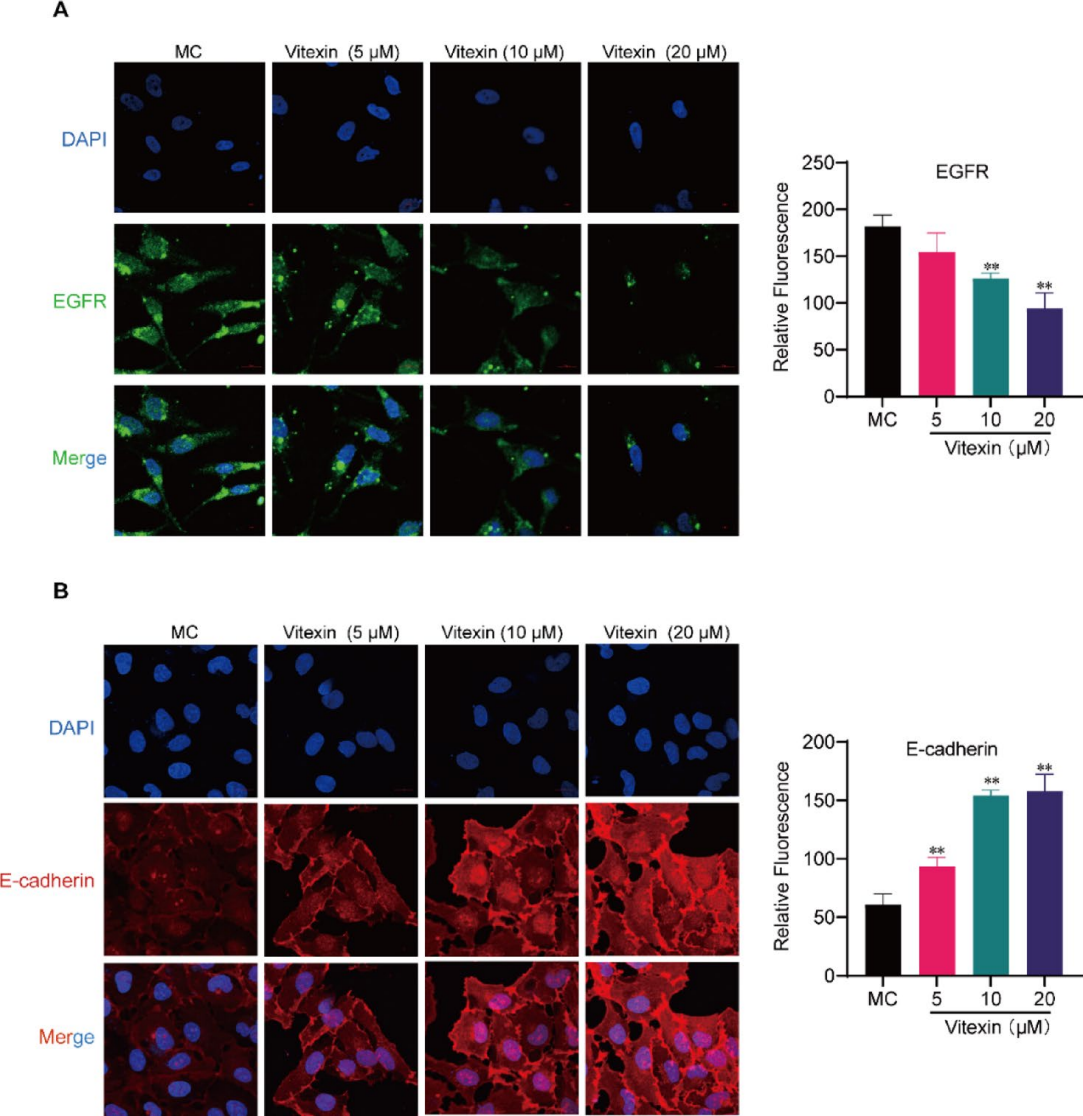
Vitexin promotes epithelial phenotypes and modulates EGFR-associated signaling in MC cells. (**A**) Representative immunofluorescence images showing EGFR expression in MC cells treated with vitexin. (Scale bar = 50 μm). (**B**) Representative immunofluorescence images showing E-cadherin expression in MC cells treated with vitexin. Data are presented as mean ± SD. (n = 3). *p < 0.05, **p < 0.01, *vs.* the MC group.

As shown in Fig. 7A, the expression and membrane localization of the oncogenic driver EGFR were significantly altered upon Vitexin treatment. In the MC model group, strong green fluorescence for EGFR was observed, predominantly localized at the cell membrane. In contrast, Vitexin treatment induced a concentration-dependent attenuation of EGFR green fluorescence intensity. Notably, in the high-dose group (20 µM), the fluorescence signal was not only markedly weakened but also became diffuse and dispersed within the cytoplasm, indicating a substantial downregulation of EGFR expression and a disruption of its characteristic membrane localization.

Conversely, as illustrated in Fig. 7B, Vitexin treatment remarkably enhanced the expression and restored the membrane localization of E-cadherin, a pivotal epithelial marker and tumor suppressor. In the MC group, red fluorescence for E-cadherin fainted and exhibited a punctate or cytoplasmic distribution. After treatment with Vitexin, a concentration-dependent augmentation of the red, fluorescent signal was evident. Most importantly, in the Vitexin (20 µM) group, a bright and continuous linear red fluorescence pattern outlining the cell membrane was clearly visible, demonstrating the potent ability of Vitexin to promote the re-expression and correct membrane assembly of E-cadherin.

In conclusion, the immunofluorescence results suggest that vitexin reduces EGFR expression and alters its membrane localization while concurrently enhancing the expression and membrane localization of E-cadherin. These findings indicate that vitexin may inhibit the migration and invasion of MC cells by modulating EGFR-associated signaling and reversing EMT-related phenotypes.

## Discussion

This study provides supportive evidence for the potential of vitexin in the intervention of PLGC. By integrating network pharmacology, molecular docking, molecular dynamics simulation, and cellular experiments, we found that vitexin exerts inhibitory effects on PLGC-related cellular phenotypes through a multi-target mode of action. Among the predicted candidates, EGFR emerged as a putative and functionally relevant target supported by both computational and cellular evidence.

The initial network analysis revealed that vitexin interacts with 33 common targets shared between PLGC-related disease pathways and the pharmacological activity of vitexin itself. This multi-target nature underscores the complexity and therapeutic potential of vitexin in managing PLGC. Importantly, STRING-based protein–protein interaction (PPI) analysis confirmed the dense interconnections between these targets, signifying their cooperative roles in driving disease progression. Among these targets, CCND1 emerged as a core node, making it a prime candidate for further mechanistic investigation. The cell cycle pathway, along with several other cancer-related pathways, was found to be significantly enriched, reinforcing the idea that vitexin likely exerts its effects by modulating key proteins involved in cell proliferation and survival.

Although CCND1 was identified as a central hub gene in the PPI network, EGFR was prioritized for further validation in the present study for several reasons. First, EGFR functions as an upstream regulator with broader relevance to proliferation, migration, and epithelial phenotypic alteration. Second, EGFR showed favorable docking performance and was further supported by molecular dynamics simulation. Therefore, EGFR was selected as the primary candidate for downstream validation in this study, although the potential contribution of CCND1 should not be overlooked.

The docking studies provided structural insights suggesting that vitexin may interact favorably with several pivotal cancer-related proteins, including EGFR, CCND1, and ABL1. Together with the MD simulation and immunofluorescence findings, these results support the view that EGFR may represent a putative and functionally relevant target of vitexin in PLGC. However, because no direct biochemical target-engagement assay was performed in the present study, the interaction between vitexin and EGFR should be interpreted cautiously rather than as definitive target validation.

To further assess whether EGFR could serve as a plausible functional target of vitexin, we evaluated the EGFR–vitexin complex using molecular dynamics simulation. The complex remained globally stable over the 100-ns simulation, with preserved protein compactness and limited fluctuations in solvent exposure. These results provided additional structural support for the stability of the EGFR–vitexin interaction and further supported the possible involvement of EGFR-associated signaling in the observed cellular effects.

At the cellular level, our findings showed that vitexin significantly inhibits cell proliferation, migration, and invasion in the MNNG-induced MC cell model, a commonly used in vitro model relevant to gastric precancerous transformation. Vitexin treatment led to a dose-dependent decrease in cell viability, with a dose inhibition rate of approximately 50% observed at 20 μM, suggesting its potential for slowing the progression of PLGC. This finding highlights the effectiveness of vitexin in modulating cell viability and supports further investigation into its application for precancerous gastric lesions. Additionally, the wound healing and transwell assays further corroborated that vitexin reduces the migratory and invasive capacity of MC cells, providing evidence for its inhibitory effects on PLGC-associated malignant phenotypes. Nevertheless, this model represents a simplified in vitro system and cannot fully reproduce the complex tissue microenvironment and histopathological heterogeneity of PLGC in vivo.

The molecular basis of vitexin’s effects in PLGC appears to involve coordinated modulation of EGFR-associated signaling and epithelial phenotype-related changes. In our cellular assays, vitexin reduced EGFR abundance and altered its membrane distribution, while simultaneously restoring E-cadherin expression and membrane localization. These findings suggest that vitexin may promote the recovery of epithelial phenotypes in MC cells and thereby contribute to the inhibition of migration and invasion. However, the downstream signaling events of EGFR were not directly examined in the present study. Therefore, the current data do not establish the activation or inhibition status of specific downstream pathways, and further investigation will be required to clarify the precise signaling cascade involved.

Overall, these findings suggest that vitexin suppresses PLGC-associated malignant phenotypes through multi-level regulation, with EGFR-associated signaling and epithelial phenotype-related changes likely representing important components of this process. Future studies should validate the target engagement of vitexin toward EGFR (e.g., biochemical binding/kinase assays) and establish efficacy in appropriate in vivo models to further assess its translational potential.

## Conclusion

In summary, our data suggest that vitexin suppresses PLGC-associated malignant phenotypes through multi-target regulation. Among the identified candidates, EGFR emerged as a putative and functionally relevant target supported by computational and cellular evidence, while modulation of EGFR-associated signaling together with restoration of E-cadherin may contribute to EMT suppression.

## Data Availability

All data are available from the corresponding author upon reasonable request.

## Abbreviations

CCK-8: Cell counting kit-8
DMSO: Dimethyl sulfoxide
EGFR: Epidermal growth factor receptor
EMT: Epithelial-mesenchymal transition
GES-1 cells: Human gastric mucosal epithelial cell lines
MC: MNNG-induced model cells
MNNG: N-Methyl-N'-nitro-N-nitrosoguanidine
PLGC: Precancerous lesions of gastric cancer
PPI: Protein–protein interaction
KEGG: Kyoto encyclopedia of genes and genomes
GO: Gene ontology
PI3K–Akt: Phosphoinositide 3-kinase/protein kinase B
